# Eptinezumab treatment initiated during a migraine attack is associated with meaningful improvement in patient-reported outcome measures: secondary results from the randomized controlled RELIEF study

**DOI:** 10.1186/s10194-021-01376-7

**Published:** 2022-02-07

**Authors:** Peter McAllister, Paul K. Winner, Jessica Ailani, Dawn C. Buse, Richard B. Lipton, George Chakhava, Mette Krog Josiassen, Annika Lindsten, Lahar Mehta, Anders Ettrup, Roger Cady

**Affiliations:** 1 New England Institute for Neurology and Headache, Stamford, CT USA; 2 Palm Beach Headache Center, West Palm Beach, FL USA; 3 Neurology Research Institute Palm Beach, West Palm Beach, FL USA; 4 Premiere Research Institute, West Palm Beach, FL USA; 5 Palm Beach Neurology, West Palm Beach, FL USA; 6 Nova Southeastern University, Fort Lauderdale, FL USA; 7 Department of Neurology, Georgetown University Hospital, Washington, DC USA; 8 Albert Einstein College of Medicine, Bronx, NY USA; 9 Vector Psychometric Group, LLC, Chapel Hill, NC USA; 10 Multiprofile Clinic Consilium Medulla, Georgian Association of Medical Specialties, David Tvildiani Medical University, Tbilisi, Georgia; 11 H. Lundbeck A/S, Copenhagen, Denmark; 12 Lundbeck Seattle BioPharmaceuticals, Inc., Bothell, WA USA; 13 Lundbeck LLC, Deerfield, IL USA

**Keywords:** Migraine, Eptinezumab, CGRP, MBS

## Abstract

**Background:**

Demonstrating therapeutic value from the patient perspective is important in patient-centered migraine management. The objective of this study was to investigate the impact of eptinezumab, a preventive migraine treatment, on patient-reported headache impact, acute medication optimization, and perception of disease change when initiated during a migraine attack.

**Methods:**

RELIEF was a randomized, double-blind, placebo-controlled trial conducted between 2019 and 2020 in adults with ≥1-year history of migraine and 4–15 migraine days per month in the 3 months prior to screening. Patients were randomized (1:1) to a 30-min infusion of eptinezumab 100 mg or placebo within 1–6 h of a qualifying migraine attack onset. The 6-item Headache Impact Test (HIT-6) and 6-item Migraine Treatment Optimization Questionnaire (mTOQ-6) were administered at baseline and week 4, and the Patient Global Impression of Change (PGIC) at week 4. A post hoc analysis of these measures was conducted in patients who reported headache pain freedom at 2 h after infusion start.

**Results:**

Of 480 patients enrolled and treated, 476 completed the study and are included in this analysis. Mean baseline HIT-6 total scores indicated severe headache impact (eptinezumab, 65.1; placebo, 64.8). At week 4, the eptinezumab-treated group demonstrated clinically meaningful improvement in HIT-6 total score compared with placebo (mean change from baseline: eptinezumab, − 8.7; placebo, − 4.5; mean [95% CI] difference from placebo: − 4.2 [− 5.75, − 2.63], *P* < .0001), with greater reductions in each item score vs placebo (*P* < .001 all comparisons). Change in HIT-6 total score in the subgroup with 2-h headache pain freedom was − 13.8 for the eptinezumab group compared with − 4.9 for the placebo group. mTOQ-6 total score mean change from baseline favored eptinezumab (change, 2.1) compared with placebo (1.2; mean [95% CI] difference: 0.9 [0.3, 1.5], *P* < .01). More eptinezumab-treated patients rated PGIC as much or very much improved than placebo patients (59.3% vs 25.9%).

**Conclusions:**

When administered during a migraine attack, eptinezumab significantly improved patient-reported outcomes after 4 weeks compared with placebo, with particularly pronounced effects in patients reporting headache pain freedom at 2 h after infusion start.

**Trial registration:**

ClinicalTrials.gov Identifier: NCT04152083. November 5, 2019.

**Supplementary Information:**

The online version contains supplementary material available at 10.1186/s10194-021-01376-7.

## Background

Migraine is a highly prevalent neurological disease characterized by attacks of moderate to severe headache associated with physiological disruptions of neurological, gastrointestinal, and sensory function [1]. Migraine-related disability impacts not only the individual with migraine, but their families, workplace, and the healthcare system [2–5]. In addition, migraine has negative impact relating to missing important events, commitment avoidance, and feelings of guilt regarding the effect on family [6, 7]. Migraine is more common in women and most prevalent between the ages of 20–40 years, amplifying its impact on family and career development [3, 8]. As migraine frequency increases, so does its impact on functionality, overall disability, and productivity [5, 9]. Patients with migraine balance lifestyle changes and trigger management with acute and preventive treatments taken to stop an attack or reduce the overall frequency of attacks [10–12]. In clinical trials for acute and preventive treatment of migraine, various endpoints and patient-reported outcome measures (PROMs) are used to evaluate efficacy [13, 14].

Eptinezumab, a humanized monoclonal antibody, is approved for the prevention of migraine in adults [15]. As an intravenous (IV) infusion with 100% bioavailability, eptinezumab has a T_max_ of 30 min with rapid, high-affinity binding to calcitonin gene-related peptide [16]. In phase 3 studies in patients with episodic migraine [17] and chronic migraine [18], eptinezumab 100 mg and 300 mg met the primary efficacy endpoint, significantly reducing mean monthly migraine days (MMDs) over weeks 1–12. In RELIEF, a phase 3 clinical trial in patients with migraine, eptinezumab 100 mg demonstrated efficacy in relieving headache pain and migraine-associated symptoms within 2 h of infusion start and reducing acute medication use compared with placebo when administered during a migraine attack [19].

The objective of this secondary analysis was to evaluate the impact of treatment in patients from the RELIEF study on PROMs captured 4 weeks after eptinezumab treatment initiated during a migraine attack.

## Methods

### Study design and patients

The RELIEF study (NCT04152083), including detailed methods, was previously described [19]. This was a 4-week, phase 3, parallel-group, double-blind, placebo-controlled clinical trial in which patients received IV eptinezumab 100 mg or placebo over 30 min within 1–6 h of a qualifying migraine attack (day 0). The use of rescue medication (any acute medication to treat migraine or migraine-associated symptoms) was not permitted in the 24-h period prior to receiving study treatment or within 2 h of infusion start. Only after 2 h post infusion start were patients permitted to use rescue medication.

RELIEF was conducted between November 2019 and July 2020 at 42 sites in the United States and 5 sites in the country of Georgia. The study was approved by a centralized institutional review board (or independent ethics committee at each study site, if required), with written informed consent obtained for each participant prior to the study’s initiation.

Eligible patients were 18–75 years of age with ≥1-year history of migraine (defined by the International Classification of Headache Disorders, 3rd edition [ICHD-3] criteria) [1], with onset of first migraine ≤50 years of age. All patients were required to have experienced migraine on 4–15 days per month in the 3 months prior to screening (screening occurred up to 8 weeks before dosing) to ensure that only patients eligible for preventive treatment were enrolled. Patients were required to have typical migraine attacks (4–72 h untreated), with headache pain of moderate to severe intensity, migraine-associated features, and a most bothersome symptom (MBS) of nausea, photophobia, or phonophobia. Additionally, patients were required to have a history of active or previous triptan use for migraine to help ensure a migraine diagnosis. Patients could not receive any monoclonal antibody treatment for any reason within 6 months prior to screening or any experimental, unregistered therapy within 30 days or 5 plasma half-lives (whichever was longer) prior to screening.

### Patient-reported outcome measures

This report focuses on the 6-item Headache Impact Test (HIT-6) [20],6-item Migraine Treatment Optimization Questionnaire (mTOQ-6) [21], and Patient Global Impression of Change (PGIC) [22]. HIT-6 and mTOQ-6 outcomes were captured at the screening visit and 4 weeks after infusion, with PGIC captured at week 4. Site staff reviewed questionnaires for completeness and clarity, and asked patients to complete any unanswered questions prior to patients leaving the clinic; the HIT-6 or mTOQ-6 total score was treated as missing if a patient response was missing the answer to ≥1 questions.

HIT-6, as detailed in Additional file 1, Table 1, measures the impact on the ability to function normally in daily life when a headache occurs [20]. It is a 6-question, Likert-type, self-reporting questionnaire with each question scored as never = 6, rarely = 8, sometimes = 10, very often = 11, and always = 13. The HIT-6 total score is calculated from summing individual items (score range of 36–78 points), with score ranges representing the following burdens of migraine: severe impact = ≥60, substantial impact = 56–59, some impact = 50–55, and little to no impact = ≤49.

The mTOQ-6 assesses the optimization of acute treatment in persons with migraine [21]. The mTOQ-6 is a 6-question, Likert-type, self-reporting questionnaire, detailed in Additional file 1, Table 1, with each item scored as never = 1, rarely = 2, less than half the time = 3, or half the time or more = 4. The mTOQ-6 total score is calculated by summing the score for each individual question (score range of 6–24 points), with a higher score indicating better treatment optimization.

The PGIC includes a single question concerning the subject’s impression of the change in the severity of their illness since the start of the study [22]. Patients were asked, *“Since receiving study drug in this study, how would you describe the change (if any) in activity limitations, symptoms, emotions, and overall quality of life as related to your migraine?”* Answers were categorized into one of 7 categories: *“very much improved”, “much improved”, “minimally improved”, “no change”, “minimally worse”, “much worse”,* or *“very much worse”.* At the week 4 visit, patients were asked to review brief instructions and then complete the assessment.

### Statistical analyses

For the HIT-6 and mTOQ-6, item and total scores were summarized by treatment group at screening and week 4 visits and change from baseline to the week 4 visit was calculated. An analysis of covariance (ANCOVA) model was used to test for a difference between treatment arms for the total score, with the model using change from baseline at week 4 as the response variable. Baseline value, treatment group, and the stratification variables of concomitant preventive migraine treatment (use vs no use) and region (North America vs Georgia) were the independent variables. A similar ANCOVA was fitted for each item of the HIT-6 and mTOQ-6. In the current analyses, the value from the week 4 visit was used for both outcome measures, regardless of whether it was an actual week 4 assessment or an early-termination assessment. The frequency distribution of PGIC responses at the week 4 visit was summarized descriptively.

An exploratory, post hoc analysis was undertaken to evaluate the ability of treatment to provide a clinically meaningful change in HIT-6 total score and item scores. A clinically meaningful within-person improvement in HIT-6 total score was defined as a 5-point or greater decrease, in line with the 2019 American Headache Society (AHS) position statement [23]. A further analysis based on a categorical HIT-6 analysis in patients with chronic migraine was also conducted [24], where a clinically meaningful within-person improvement was defined as a ≥ 6-point decrease in HIT-6 total score, a ≥ 1-category decrease in items 1–3, and a ≥ 2-category decrease in items 4–6.

As part of the efficacy assessment reported in Winner et al. [19], key secondary endpoints included headache pain freedom and absence of MBS at 2 h after infusion start, and additional secondary endpoints included headache pain freedom and absence of MBS at 4 h after infusion start. To evaluate the potential clinical meaningfulness of these improvements for changes in PROMs, a post hoc subgroup analysis of HIT-6 total score was conducted in patients who did and did not achieve these secondary endpoints. In addition to evaluating HIT-6 total score, the percentage of patients reporting a new migraine within the study period was calculated in each subgroup; the incidence of a new migraine was captured in an electronic diary from day 3 following treatment until a new migraine was reported (up to day 28).

## Results

### Patients

Of the 480 patients randomized to treatment, 235/238 (98.7%) patients assigned to eptinezumab and 241/242 (99.6%) patients assigned to placebo completed the study. Baseline demographics and characteristics have been previously reported [19], with similarity between eptinezumab and placebo groups (Table 1).

**Table 1:** Overview of demographics and baseline headache characteristics

	Eptinezumab (*N* = 238)	Placebo (*N* = 242)
**Age** (years), mean (SD)	44.9 (12.0)	44.1 (12.1)
**Sex: Female**, no. (%)	202 (84.9)	201 (83.1)
**Race**, no. (%)		
White	200 (84.0)	213 (88.0)
Black or African American	30 (12.6)	19 (7.9)
Other^a^	8 (3.4)	10 (4.1)
**History of chronic migraine**, no. (%)^b^	25 (10.5)	27 (11.2)
**Monthly migraine days**, mean (SD)^c^	7.2 (2.7)	7.2 (2.6)
**Duration of migraine prior to infusion start** (hours), mean (SD)^d^	3.7 (1.0)	3.7 (1.0)
**Severity of headache pain**, no. (%)		
Moderate	110 (46.2)	117 (48.3)
Severe	128 (53.8)	123 (50.8)

*SD* standard deviation

^a^Other includes Asian, American Indian or Alaska Native, Native Hawaiian or other Pacific Islander, other, and multiple

^b^Migraine history was collected at the screening visit by the investigator through medical records; if medical records could not be obtained, history was confirmed via patient interview in order to obtain sufficient information to confirm all eligibility criteria are met

^c^Patients self-reported the average number of monthly migraine days over the 3 months prior to screening

^d^Duration was calculated as the difference between the study drug infusion start date and time and the day 1 headache start date and time

### 6-item Headache Impact Test

**Fig. 1:**
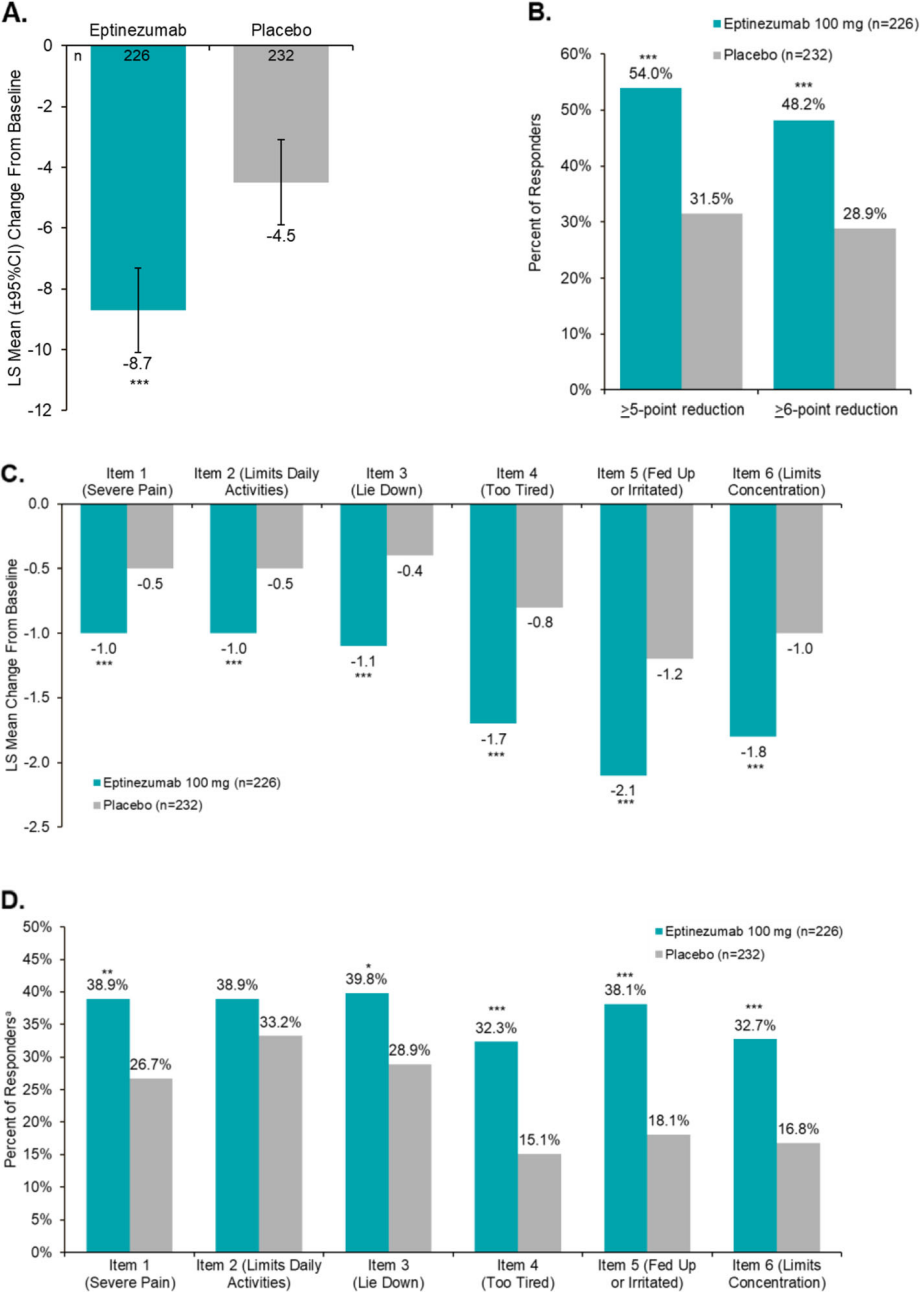
Effects of Eptinezumab vs Placebo on HIT-6–Related Outcomes. **A** Least Squares (LS) Mean Change From Baseline at Week 4; **B** Responder Rates at Week 4 for HIT-6 Total Score; **C** LS Mean Change From Baseline in HIT-6 Item Scores at Week 4; **D** Responder Rates for HIT-6 Item Scores at Week 4. CI, confidence interval; HIT-6, 6-item Headache Impact Test; LS, least square. *P < .05, **P < .01, ***P < .001 vs placebo. Limited to patients with both baseline and post-baseline HIT-6 data. ^a^For items 1–3, a responder was defined as a patient with an improvement of ≥1 category; for items 4–6, a responder was defined as a patient with an improvement of ≥2 categories [24]. Error bars represent 95% CI

At baseline, the mean HIT-6 total score was 64.9 and was similar across treatment groups (eptinezumab, 65.1; placebo, 64.8). At week 4, the least squares (LS) mean (95% CI) change from baseline in HIT-6 total score was − 8.7 (− 10.1, − 7.3) with eptinezumab 100 mg vs − 4.5 (− 5.9, − 3.1) with placebo (*P <* .0001) (Table 2 and Fig. 1A). Using the 5-point reduction threshold [23] to determine a clinically meaningful change in HIT-6 total score, a clinical response was achieved by 54.0% (*n* = 122) of patients treated with eptinezumab compared with 31.5% (*n* = 73) of patients treated with placebo (*P <* .0001) (Fig. 1B). When analyzed using the 6-point reduction threshold [24], a clinically meaningful change in HIT-6 total score was achieved by 48.2% (*n* = 109) of eptinezumab-treated patients compared with 28.9% (*n* = 67) of placebo patients (*P <* .0001) (Fig. 1B).

**Table 2:** Patient-reported outcomes at baseline and week 4

	Eptinezumab	Placebo
**HIT-6 total score**, n^a^	226	232
Baseline, mean (SD)	65.1 (4.97)	64.8 (5.01)
Week 4, mean (SD)	57.0 (9.74)	61.1 (7.83)
Change from baseline, LS mean (95% CI)	−8.7 (− 10.1, − 7.3)	−4.5 (− 5.9, − 3.1)
Difference from placebo, LS mean (95% CI)^b^	− 4.2 (− 5.7, − 2.6)	
*P*-value vs placebo	< .0001	
**mTOQ-6 total score**, n^a^	226	231
Baseline, mean (SD)	18.1 (4.05)	18.6 (4.20)
Week 4, mean (SD)	20.1 (4.21)	19.6 (4.22)
Change from baseline, LS mean (95% CI)	2.1 (1.5, 2.6)	1.2 (0.7, 1.7)
Difference from placebo, LS mean (95% CI)^b^	0.9 (0.3, 1.5)	
*P*-value vs placebo	.0053	
**PGIC rating at week 4**, n^a^	226	232
Very much improved, n (%)	56 (24.8)	29 (12.5)
Much improved, n (%)	78 (34.5)	31 (13.4)
Minimally improved, n (%)	54 (23.9)	58 (25.0)
No change, n (%)	35 (15.5)	109 (47.0)
Worse, n (%)^c^	3 (1.3)	5 (2.1)

*CI* confidence interval, *HIT-6* 6-item Headache Impact Test, *LS* least squares, *mTOQ-6* 6-item Migraine Treatment Optimization Questionnaire, *PGIC* Patient Global Impression of Change, *SD* standard deviation

^a^Limited to patients with both baseline and post-baseline data. All *P*-values are descriptive

^b^The estimated mean, mean difference from placebo, and 95% CI are from an analysis of covariance adjusted for baseline value and stratification factors of concomitant treatment and region

^c^Worse includes “*minimally worse*”, “*much worse*”, and “*very much worse*”

At baseline, HIT-6 item scores were similar across treatment arms (see Additional file 1, Table 2) and reflective of a population with frequent migraine attacks [25]. The LS mean change from baseline at week 4 for HIT-6 items 1–3 was approximately − 1.0 with eptinezumab, compared with approximately − 0.5 with placebo across items (*P <* .001, all items 1–3; Fig. 1C). In the analysis of HIT-6 items 4–6, the LS mean change from baseline at week 4 ranged from − 1.7 to − 2.1 with eptinezumab and from − 0.8 to − 1.2 with placebo (*P <* .0001, all items 4–6; Fig. 1C). For each HIT-6 item, 30%–40% of eptinezumab-treated patients achieved a clinical response; for patients who received placebo, 26.7%–33.2% achieved response on items 1–3 and 15.1%–18.1% achieved response on items 4–6 (Fig. 1D) (*P <* .05 for all except item 2 [limits daily activities]).

**Fig. 2:**
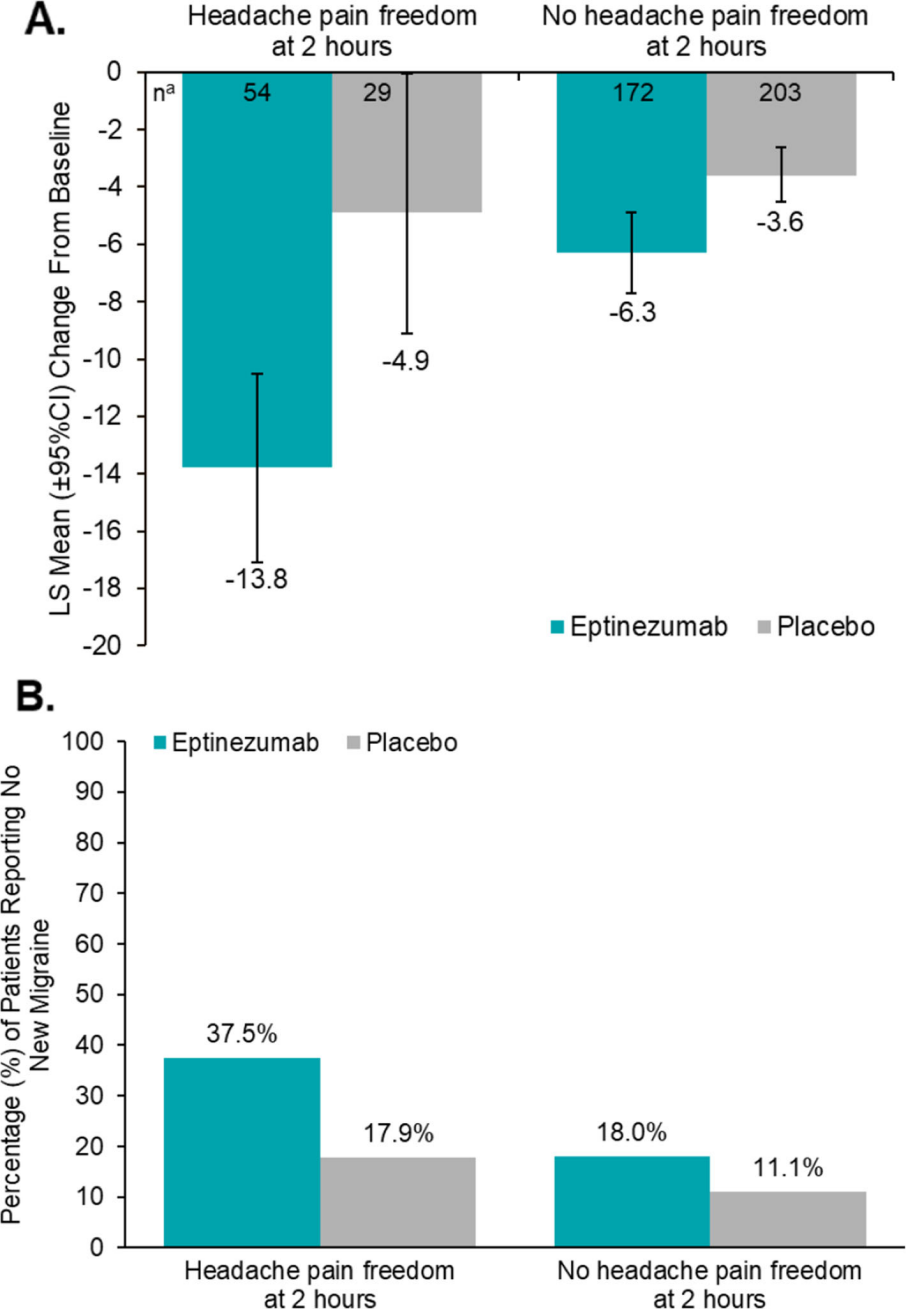
Relationship of 2-h Pain Freedom to Change in HIT-6 and Occurrence of Subsequent Migraine. **A** Mean Change From Baseline to Week 4 in HIT-6 Total Score and **B** Percent of Patients Without a New Migraine Occurring With or Without Headache Pain Freedom at 2 Hours After Infusion Start. CI, confidence interval; HIT-6, 6-item Headache Impact Test; LS, least square. ^a^Limited to patients with both baseline and post-baseline HIT-6 data Error bars represent 95% CI

In patients reporting headache pain freedom at 2 h, the mean HIT-6 total score at baseline was 67.3 for the eptinezumab group (*n* = 54) and 64.5 for the placebo group (*n* = 29). At week 4, the mean change from baseline in HIT-6 total score (95% CI) in the eptinezumab-treated patients who achieved 2-h pain freedom was − 13.8 (− 17.1, − 10.5; *n* = 54) compared with − 4.9 in patients receiving placebo who were pain free at 2 h (− 9.1, − 0.6; *n* = 29; Fig. 2A). Among 2-h pain freedom responders (eptinezumab, 56/238; placebo, 29/242), a larger proportion of the eptinezumab group (37.5%) did not experience a new migraine attack within the 4-week follow-up period of the study compared with patients in the placebo group (17.9%) (Fig. 2B). Similar benefits of early response were observed in patients experiencing absence of (MBS at 2 h, headache pain freedom at 4 h, and absence of MBS at 4 h (see Additional file 1, Table 3).

### 6-item Migraine Treatment Optimization Questionnaire

**Fig. 3:**
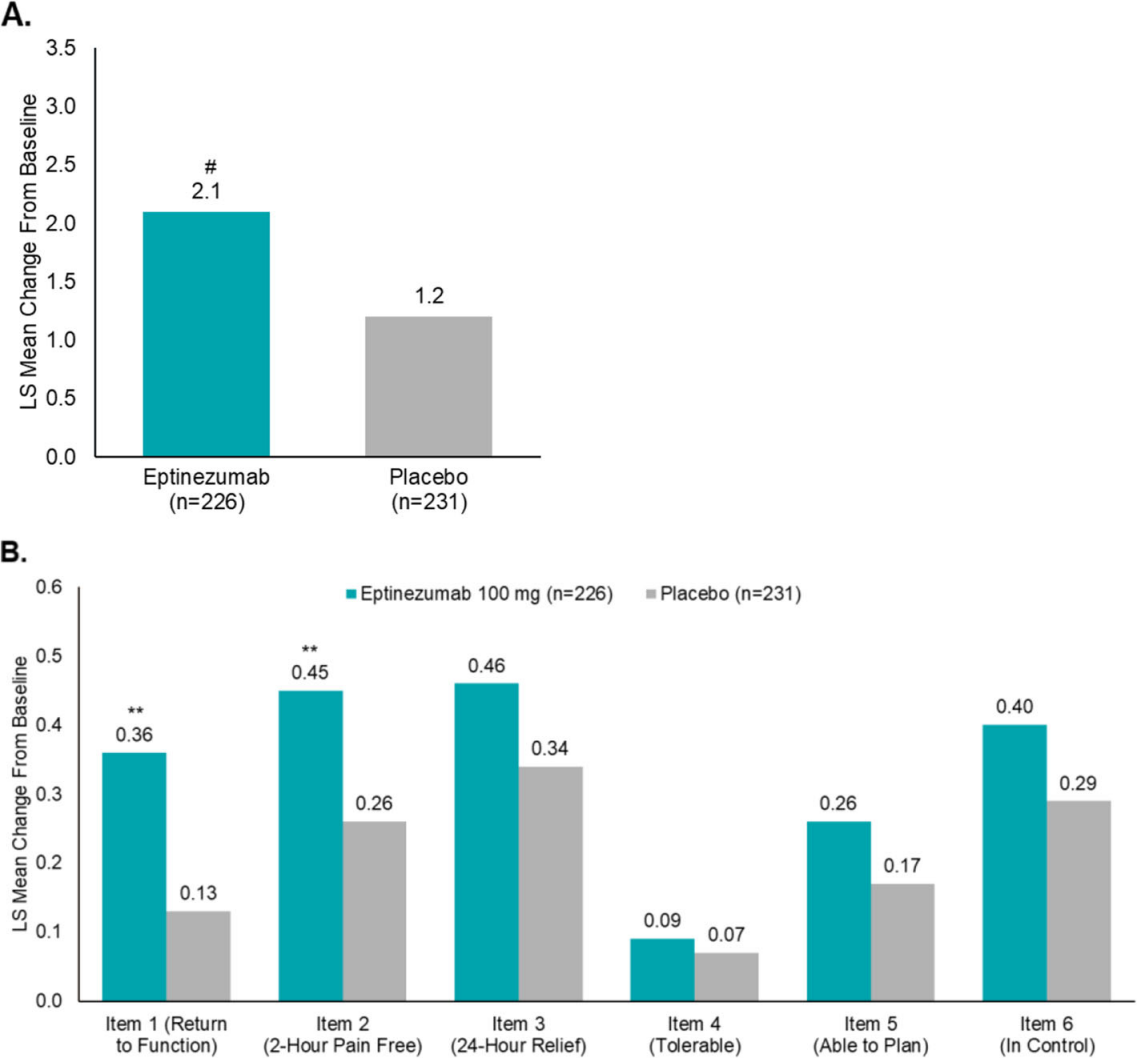
mTOQ-6 Mean Change From Baseline to Week 4. **A** Mean change from baseline to week 4 in mTOQ-6 total score and **B** in mTOQ-6 item scores. mTOQ-6, 6-item Migraine Treatment Optimization Questionnaire. ^a^Limited to patients with both baseline and post-baseline mTOQ-6 data. **P < .01, #*P* = .0053 vs placebo

At baseline, the mTOQ-6 total score was similar across both treatment arms (eptinezumab, 18.1; placebo, 18.6) and reflective of patients diagnosed with episodic migraine [21]. At week 4, the LS mean (95% CI) change from baseline in mTOQ-6 total score (Fig. 3A) was 2.1 (1.5, 2.6) in patients treated with eptinezumab, compared with 1.2 (0.7, 1.7) in patients receiving placebo (*P <* .01). For patients reporting headache pain freedom at 2 h post infusion start, the improvement in mTOQ-6 was 3.1 (1.7, 4.5) in patients treated with eptinezumab, compared with 1.8 (0.5, 3.2) in patients receiving placebo. Analysis of mTOQ-6 item scores was again similar across treatment groups at baseline (see Additional file 1, Table 4), with analysis of the change from baseline at week 4 resulting in greater improvements for mTOQ-6 item 1 (quickly return to function) and item 2 (pain-free within 2 h) when compared with placebo (*P <* .01) (Fig. 3B).

### Patient Global Impression of Change

Analysis of the PGIC (Table 2) identified more than twice as many eptinezumab-treated patients reporting “much” or “very much” improvement in disease status compared with placebo (59.3% vs 25.9%, respectively). Overall, clinical improvement was reported by 83.2% of eptinezumab-treated patients compared with 50.9% of placebo patients. No change in disease status was reported by 15.5% of eptinezumab-treated patients compared with 47.0% of placebo patients.

## Discussion

In the RELIEF study, patients receiving eptinezumab, a preventive migraine treatment, during a migraine attack achieved a faster time to headache pain freedom and absence of MBS compared with patients receiving placebo [19]. Patients with migraine who were administered eptinezumab during a migraine attack demonstrated greater improvements in the PROMs of HIT-6, mTOQ-6, and PGIC compared to those receiving placebo.

We also compared the benefits of treatment on these PROMs among those who did and did not achieve freedom from pain and MBS 2 h post treatment. A favorable short-term (2-h) response to treatment was associated with a favorable response to PROMs at 4 weeks. This short-term response, which occurred more often in eptinezumab-treated patients, resulted in the reduction of overall headache-related impact (HIT-6) as well as a reduction in the potential for experiencing another migraine attack during the study period. For patients with frequent migraine attacks (4–15 days per month), experiencing both early (2-h) onset of treatment effect and a decreased chance of having another migraine attack for the 4 weeks following infusion could underly the robust improvement observed on the HIT-6 in this population. Prior work has not evaluated the association between acute benefits of a preventive treatment and longer-term benefits; further research is needed to determine if acute treatment success for a single attack might reduce headache frequency on a longer term basis. It should be noted that, as a preventive treatment, eptinezumab is administered every 12 weeks, with a previous analysis showing statistically significant preventive efficacy relative to placebo beginning on post-treatment day 1 and extending through the 12-week dosing interval [26]; therefore, some of the effect on week 4 outcomes was likely related to the rapid onset of preventive efficacy with eptinezumab.

These findings confirm the substantial burden that migraine places on patients and identified a treatment benefit in favor of eptinezumab compared with placebo in relieving the disease burden in patients experiencing up to 15 migraine days per month. This treatment benefit, as demonstrated by improvements in HIT-6 (in the total population as well as the more pronounced improvements in patients with headache pain freedom 2 h after start of eptinezumab infusion), was clinically meaningful according to the guidance from the AHS [23] and meets the criteria established for a clinically meaningful change in patients with chronic migraine at a total score and individual item level [24]. Further, the patient-reported treatment benefits observed on the HIT-6 in this study replicate the results reported in the phase 3 PROMISE-2 study in patients with chronic migraine [18, 27], underpinning the importance of evaluating and monitoring the impact of migraine with the HIT-6 in all patients.

The impact of eptinezumab treatment on the PGIC highlights the importance of evaluating the patient’s perception of benefits that are directly associated with treatment. The present analyses identified that more than twice as many patients treated with eptinezumab reported *“much improved”* or *“very much improved”* on the PGIC compared to placebo, indicating a high degree of patient satisfaction associated with eptinezumab. Studies in migraine patients have found a direct correlation between PGIC improvement and a reduction in impact on the HIT-6 [24], and while not tested in this patient population, it is expected that similar results would be observed, further supporting the clinical meaningfulness of the PGIC results. Of particular interest is the greater number of patients indicating “*much improved”* or *“very much improved”* for those who reported headache pain freedom at 2 h. In this population, the PGIC response at week 4 highlights the importance of an early “patient-perceived” response to treatment in the long-term management of migraine.

Patients responded better on mTOQ-6 at 4 weeks after treatment with eptinezumab compared to those who received placebo, based on change from baseline in mTOQ-6 total score, suggesting that eptinezumab may work synergistically with acute treatments used to treat future migraines. Patients treated with eptinezumab reported a better score compared to patients receiving placebo on mTOQ-6 items 1 and 2 (return to normal and pain free within 2 h), indicating they were more likely to achieve 2-h pain-free outcomes when treating future migraine attacks and able to function better. The lack of difference between eptinezumab-treated and placebo patients on items 5 and 6 (planning activities and expecting no disruptions due to migraine) indicates that although patients report favorably on returning to normal, it may take longer than 4 weeks for patients to feel in control of managing their migraine, especially given that most patients were experiencing fewer migraine attacks at week 4 than they did at baseline. Given patients were administered eptinezumab in an acute treatment setting, they had no expectations that their usual acute medication usage would be impacted; therefore, findings that eptinezumab provided a greater ability to return to normal functioning (item 1), be pain-free at 2 h after acute treatment (item 2), and had no impact on the tolerability of current medication (item 4) are consistent with a potential of eptinezumab to work synergistically with acute migraine treatments.

### Limitations

Several limitations should be considered. The data for the RELIEF study were generated in the clinical trial setting; thus, participants may differ from those in general practice, potentially limiting the overall generalizability of the results. Further, patients reported a higher migraine severity and impact than previously reported in similar patient populations. Additionally, because changes in MMDs—a traditional outcome for migraine preventive treatments—were not captured over longer term (e.g. over 12 weeks) *using a daily electronic diary*, changes in PROMs could not be compared to reductions in MMDs. Finally, while PROM assessment demonstrated a clinically meaningful benefit at week 4, no PROMs were captured after 12 weeks (the approved treatment interval for eptinezumab), limiting the long-term applicability of the results since it likely takes longer than 4 weeks for patients to adjust their expectations after initiation of preventive treatment.

## Conclusions

Preventive migraine treatment with eptinezumab initiated during a migraine attack provided clinically meaningful improvements in several PROMs as early as 4 weeks after infusion. These benefits were especially noticeable in those patients who reported headache pain freedom at 2 h, highlighting the significance of experiencing the early onset of effect with eptinezumab. In addition, patients treated with eptinezumab relative to those receiving placebo reported greater effectiveness of their acute medication used after initiation during a migraine attack, findings that may raise the standards for preventive treatments and provide promising outcomes for patients suffering from migraine.

## Supplementary Information

**Additional file 1.**

## Data Availability

In accordance with EFPIA’s and PhRMA’s “Principles for Responsible Clinical Trial Data Sharing” guidelines, Lundbeck is committed to responsible sharing of clinical trial data in a manner that is consistent with safeguarding the privacy of patients, respecting the integrity of national regulatory systems, and protecting the intellectual property of the sponsor. The protection of intellectual property ensures continued research and innovation in the pharmaceutical industry. The data were collected during the study using an electronic data capture (EDC) device and reported on each patient’s individual case report form (CRF). All data reports, including the PGIC, HIT-6, and mTOQ-6 questionnaires, were documented within the EDC system, with each set of completed CRFs reviewed and verified by the site monitor and electronically signed and dated by the investigator. Deidentified data are available to those whose request has been reviewed and approved through an application submitted to https://www.lundbeck.com/global/our-science/clinical-data-sharing
