# Supplementary material for: Eptinezumab treatment initiated during a migraine attack is associated with meaningful improvement in patient-reported outcome measures: secondary results from the randomized controlled RELIEF study
Source: J Headache Pain. 2022 Feb 7;23(1):22. doi: 10.1186/s10194-021-01376-7 (PMC8903522; doi:10.1186/s10194-021-01376-7)
Supplement: Supplementary file 1 — Additional file 1. [file 10194_2021_1376_MOESM1_ESM.docx]

# ADDITIONAL FILE 1

## Table 1. HIT-6 and mTOQ-6 Questionnaires

| Item No. | Question |
| --- | --- |
| **6-Item Headache Impact Test (HIT-6)** | |
| Item 1 | When you have headaches, how often is the pain severe? |
| Item 2 | How often do headaches limit your ability to do usual daily activities including household work, work, school, or social activities? |
| Item 3 | When you have a headache, how often do you wish you could lie down? |
| Item 4 | In the past 4 weeks, how often have you felt too tired to do work or daily activities because of your headaches? |
| Item 5 | In the past 4 weeks, how often have you felt fed up or irritated because of your headaches? |
| Item 6 | In the past 4 weeks, how often did headaches limit your ability to concentrate on work or daily activities? |
| Response Options | Never, rarely, sometimes, very often, or always |
| **6-Item Migraine Treatment Optimization Questionnaire (mTOQ-6)** | |
| Item 1 | Are you able to quickly return to normal activities (ie, work, family, leisure, social activities) after taking your migraine medication? |
| Item 2 | After taking your migraine medication, are you pain free within 2 hours for most attacks? |
| Item 3 | Does one dose of your migraine medication usually relieve your headache and keep it away for at least 24 hours? |
| Item 4 | Is your migraine medication well tolerated? |
| Item 5 | Are you comfortable enough with your migraine medication to be able to plan your daily activities? |
| Item 6 | After taking your migraine medication, do you feel in control of your migraines enough so that you feel there will be no disruption to your daily activities? |
| Response Options | Never, rarely, less than half the time, or half the time or more |

## Table 2. HIT-6 Item Score at Baseline and at Week 4

|  | **Eptinezumab** (N = 226)^a^ | **Placebo** (N = 232)^a^ | **Difference**, LS mean (95% CI)^b^ | ***P* value** |
| --- | --- | --- | --- | --- |
| **HIT-6 item 1** |  |  |  |  |
| Baseline, mean (SD) | 11.0 (1.01) | 10.9 (0.87) |  |  |
| Week 4, mean (SD) | 10.1 (1.79) | 10.6 (1.29) |  |  |
| Change from baseline, LS mean (95% CI) | -1.0 (-1.3, -0.8) | -0.5 (-0.7, -0.3) | -0.5 (-0.8, -0.3) | < .001 |
| **HIT-6 item 2** |  |  |  |  |
| Baseline, mean (SD) | 10.8 (1.07) | 10.8 (1.13) |  |  |
| Week 4, mean (SD) | 9.9 (1.81) | 10.4 (1.39) |  |  |
| Change from baseline, LS mean (95% CI) | -1.0 (-1.3, -0.8) | -0.5 (-0.8, -0.3) | -0.5 (-0.8, -0.2) | < .001 |
| **HIT-6 item 3** |  |  |  |  |
| Baseline, mean (SD) | 11.5 (1.42) | 11.5 (1.38) |  |  |
| Week 4, mean (SD) | 10.6 (2.19) | 11.2 (1.60) |  |  |
| Change from baseline, LS mean (95% CI) | -1.1 (-1.4, -0.8) | -0.4 (-0.7, -0.2) | -0.7 (-1.0, -0.3) | < .001 |
| **HIT-6 item 4** |  |  |  |  |
| Baseline, mean (SD) | 10.4 (1.15) | 10.3 (1.16) |  |  |
| Week 4, mean (SD) | 8.7 (1.92) | 9.6 (1.65) |  |  |
| Change from baseline, LS mean (95% CI) | -1.7 (-2.0, -1.4) | -0.8 (-1.1, -0.5) | -0.9 (-1.2, -0.6) | < .001 |
| **HIT-6 item 5** |  |  |  |  |
| Baseline, mean (SD) | 10.7 (1.36) | 10.5 (1.56) |  |  |
| Week 4, mean (SD) | 8.8 (2.16) | 9.6 (2.10) |  |  |
| Change from baseline, LS mean (95% CI) | -2.1 (-2.4, -1.7) | -1.2 (-1.6, -0.9) | -0.8 (-1.2, -0.4) | < .001 |
| **HIT-6 item 6** |  |  |  |  |
| Baseline, mean (SD) | 10.7 (1.10) | 10.7 (1.26) |  |  |
| Week 4, mean (SD) | 9.0 (1.98) | 9.8 (1.82) |  |  |
| Change from baseline, LS mean (95% CI) | -1.8 (-2.1, -1.5) | -1.0 (-1.3, -0.7) | -0.8 (-1.2, -0.5) | < .001 |

CI, confidence interval; HIT-6, 6-item Headache Impact Test; LS, least squares; SD, standard deviation.

^a^Limited to patients with both baseline and post-baseline data.

^b^The estimated LS mean, LS mean difference from placebo, and 95% CI are from an analysis of covariance adjusted for baseline value and stratification factors of concomitant treatment and region.

## Table 3. HIT-6 Total Score and Incidence of New Migraine in Patient Subgroups Defined by Treatment Response

|  | **Responders** | | **Non-responders** | |
| --- | --- | --- | --- | --- |
|  | **Eptinezumab** | **Placebo** | **Eptinezumab** | **Placebo** |
| **Headache pain freedom at 2 hours** | | | | |
| HIT-6 total score | N = 56 | N = 29 | N = 182 | N = 213 |
| Baseline, mean (SD) | 67.3 (5.2) | 64.5 (4.8) | 64.6 (4.7) | 64.9 (5.1) |
| Week 4, mean (SD) | 53.6 (10.9) | 59.6 (9.6) | 58.1 (9.1) | 61.3 (7.6) |
| Change, mean (SD) [n] | -13.8 (12.1) [54] | -4.9 (11.2) [29] | -6.3 (9.1) [172] | -3.6 (7.1) [203] |
| New migraine, n/N (%)^a^ | 35/56 (63%) | 23/28 (82%) | 146/178 (82%) | 185/208 (89%) |
| **Absence of MBS at 2 hours** | | | | |
| HIT-6 total score | N = 132 | N = 86 | N = 106 | N = 154 |
| Baseline, mean (SD) | 65.6 (4.7) | 64.9 (5.6) | 64.7 (5.2) | 64.7 (4.6) |
| Week 4, mean (SD) | 55.0 (10.3) | 59.4 (8.5) | 59.6 (8.3) | 61.9 (7.3) |
| Change, mean (SD) [n] | -10.5 (11.5) [125] | -5.5 (9.7) [81] | -5.0 (7.9) [101] | -2.9 (6.2) [149] |
| New migraine, n/N (%)^a^ | 95/132 (72%) | 71/84 (85%) | 86/102 (84%) | 135/150 (90%) |
| **Headache pain freedom at 4 hours** | | | | |
| HIT-6 total score | N = 111 | N = 64 | N = 127 | N = 178 |
| Baseline, mean (SD) | 65.9 (4.5) [111] | 64.8 (4.7) [64] | 64.6 (5.2) [127] | 64.8 (5.1) [178] |
| Week 4, mean (SD) | 54.5 (10.1) [107] | 58.9 (8.0) [62] | 59.3 (8.8) [119] | 61.9 (7.7) [170] |
| Change, mean (SD) [n] | -11.4 (11.1) [107] | -6.0 (8.9) [62] | -5.1 (8.7) [119] | -2.9 (7.1) [170] |
| New migraine, n/N (%)^a^ | 78/111 (70%) | 51/63 (81%) | 103/123 (84%) | 157/173 (91%) |
| **Absence of MBS at 4 hours** | | | | |
| HIT-6 total score | N = 155 | N = 90 | N = 83 | N = 150 |
| Baseline, mean (SD) | 65.5 (4.3) | 64.9 (5.8) | 64.6 (5.8) | 64.7 (4.5) |
| Week 4, mean (SD) | 55.3 (10.1) | 59.7 (8.2) | 60.2 (8.3) | 61.8 (7.5) |
| Change, mean (SD) [n] | -10.2 (11.0) [147] | -5.1 (9.8) [85] | -4.2 (7.8) [79] | -3.0 (6.1) [145] |
| New migraine, n/N (%)^a^ | 113/155 (73%) | 74/88 (84%) | 68/79 (86%) | 132/146 (90%) |

HIT-6, 6-item Headache Impact Test; MBS, most bothersome symptom; SD, standard deviation.

Incidence of new migraine, as recorded in an electronic diary, beginning at least 3 days (72 hours) after dosing and within 4 weeks of treatment.

## T 4. mTOQ-6 Item Score at Baseline and at Week 4

|  | **Eptinezumab** (N = 226)^a^ | **Placebo** (N = 231)^a^ | **Difference**, LS mean (95% CI)^b^ | ***P* value** |
| --- | --- | --- | --- | --- |
| **mTOQ-6 item 1** |  |  |  |  |
| Baseline, mean (SD) | 3.0 (0.92) | 3.1 (0.91) |  |  |
| Week 4, mean (SD) | 3.3 (0.89) | 3.2 (0.95) |  |  |
| Change from baseline, LS mean (95% CI) | 0.4 (0.2, 0.5) | 0.1 (-0.0, 0.3) | 0.2 (0.1, 0.4) | .003 |
| **mTOQ-6 item 2** |  |  |  |  |
| Baseline, mean (SD) | 2.9 (0.99) | 2.9 (1.01) |  |  |
| Week 4, mean (SD) | 3.3 (0.91) | 3.1 (0.95) |  |  |
| Change from baseline, LS mean (95% CI) | 0.5 (0.3, 0.6) | 0.3 (0.1, 0.4) | 0.2 (0.1, 0.3) | .007 |
| **mTOQ-6 item 3** |  |  |  |  |
| Baseline, mean (SD) | 2.8 (0.99) | 2.8 (1.08) |  |  |
| Week 4, mean (SD) | 3.2 (0.95) | 3.1 (1.01) |  |  |
| Change from baseline, LS mean (95% CI) | 0.5 (0.3, 0.6) | 0.3 (0.2, 0.5) | 0.1 (-0.0, 0.3) | .14 |
| **mTOQ-6 item 4** |  |  |  |  |
| Baseline, mean (SD) | 3.5 (0.89) | 3.6 (0.77) |  |  |
| Week 4, mean (SD) | 3.6 (0.79) | 3.6 (0.76) |  |  |
| Change from baseline, LS mean (95% CI) | 0.1 (-0.0, 0.2) | 0.1 (-0.0, 0.2) | 0.0 (-0.1, 0.2) | .68 |
| **mTOQ-6 item 5** |  |  |  |  |
| Baseline, mean (SD) | 3.2 (0.89) | 3.2 (0.97) |  |  |
| Week 4, mean (SD) | 3.4 (0.86) | 3.3 (0.90) |  |  |
| Change from baseline, LS mean (95% CI) | 0.3 (0.1, 0.4) | 0.2 (0.0, 0.3) | 0.1 (-0.0, 0.2) | .21 |
| **mTOQ-6 item 6** |  |  |  |  |
| Baseline, mean (SD) | 2.9 (0.91) | 3.0 (0.97) |  |  |
| Week 4, mean (SD) | 3.3 (0.91) | 3.2 (0.93) |  |  |
| Change from baseline, LS mean (95% CI) | 0.4 (0.3, 0.5) | 0.3 (0.2, 0.4) | 0.1 (-0.0, 0.3) | .16 |

CI, confidence interval; LS, least squares; mTOQ-6, 6-item Migraine Treatment Optimization Questionnaire; SD, standard deviation.

^a^Limited to patients with both baseline and post-baseline data.

^b^The estimated LS mean, LS mean difference from placebo, and 95% CI are from an analysis of covariance adjusted for baseline value and stratification factors of concomitant treatment and region.
